# Manual Therapy, Core Training, and Pilates Method Interventions in Dance Rehabilitation: A Scoping Review

**DOI:** 10.3390/healthcare14070872

**Published:** 2026-03-28

**Authors:** Ioannis Tsartsapakis, Aglaia Zafeiroudi

**Affiliations:** 1 Department of Physical Education and Sport Sciences at Serres, Aristotle University of Thessaloniki, 62100 Serres, Greece; 2 Department Physical Education and Sport Science, University of Thessaly, 42100 Trikala, Greece; azafeiroudi@uth.gr

**Keywords:** manual therapy, core stability, Pilates, musculoskeletal disorders, dancers, rehabilitation, scoping review

## Abstract

**Highlights:**

**What are the main findings?**

- Manual therapy remains the most frequently documented rehabilitation approach for adult dancers, while core stability and Pilates Method interventions are now actively emerging in the clinical literature, supported by recent studies.
- Despite this progress, the available evidence remains methodologically heterogeneous and heavily reliant on case reports, though a necessary transition toward randomized controlled trials is currently underway.

**What are the implications of the main findings?**

- There is a critical need for rigorous, high-quality, and dancer-specific clinical trials to empirically validate the effectiveness of manual therapy, core stability, and Pilates Method rehabilitation.
- Developing standardized, evidence-driven clinical protocols and utilizing objective outcome measures will guide clinical decision-making, safely facilitate return to performance, and improve long-term injury outcomes in dance medicine.

**Abstract:**

Objectives: To map and synthesize the available evidence regarding the use of manual therapy, core stability training, and Pilates Method exercises in the rehabilitation of musculoskeletal conditions in adult dancers. Methods: A scoping review was conducted in accordance with the PRISMA-ScR guidelines. A systematic search across six electronic databases (PubMed, Scopus, Web of Science, SPORTDiscus, CINAHL, and PEDro) was performed. Study eligibility was strictly guided by the Population–Concept–Context (PCC) framework. Two independent reviewers screened the literature and extracted data. Results: A total of 16 studies met the inclusion criteria, encompassing randomized controlled trials, intervention studies, and case-level reports or series. Interventions primarily addressed chronic ankle instability, low back pain, and post-surgical rehabilitation. Results indicate that while individualized manual therapy and Pilates Method protocols are widely utilized in dance medicine, high-level evidence remains limited. Conclusions: Integrated rehabilitation approaches combining manual therapy with motor control exercises (such as the Pilates Method and core training) appear to have potential utility in supporting the safe return of dancers to performance. However, the current literature is heavily reliant on observational and case-driven evidence, highlighting the need for robust clinical trials to establish standardized, dance-specific rehabilitation guidelines.

## 1. Introduction

### 1.1. Musculoskeletal Disorders in Dancers and the Need for Targeted Rehabilitation

Musculoskeletal disorders (MSDs) represent one of the most significant causes of pain, disability, and functional limitation, affecting an estimated 1.69 billion individuals worldwide [1]. These conditions encompass a broad spectrum of inflammatory, degenerative, and mechanical pathologies involving muscles, tendons, ligaments, joints, and related soft tissues. Common musculoskeletal disorders include low back pain, osteoarthritis, rheumatoid arthritis, tendinopathies, sprains, strains, and overuse injuries [2,3]. In performing artists and dancers, MSDs are particularly prevalent due to the high physical demands, repetitive loading, extreme ranges of motion, and aesthetic requirements of dance performance. Epidemiological studies consistently show that dancers experience high rates of hip pain, low back pain, tendinopathy, and stress-related injuries, often exceeding those observed in many athletic populations [4,5].

The rehabilitation needs of dancers are unique because their injuries frequently arise from complex biomechanical patterns, technique-specific demands, and prolonged training exposure. Effective management requires early diagnosis, individualized treatment planning, and interventions tailored to the stage of tissue healing and the functional requirements of dance [6,7]. Multidisciplinary and multimodal rehabilitation programs have demonstrated effectiveness in improving pain, function, and return-to-activity outcomes in MSDs, highlighting the importance of integrating physical therapy, manual therapy, exercise-based interventions, and psychosocial support [8,9]. For dancers, rehabilitation must also address movement quality, neuromuscular control, proprioception, and the restoration of technique-specific motor patterns [10,11].

### 1.2. Key Rehabilitation Concepts: Manual Therapy and Pilates Method Interventions

Within the specialized field of dance medicine, non-pharmacological interventions form the cornerstone of conservative management. At the forefront of these modalities are manual therapy and Pilates. In the context of this review, manual therapy is defined as a specialized area of physical therapy that utilizes skilled, hands-on techniques to treat musculoskeletal pain and dysfunction. This includes joint mobilization, joint manipulation, soft tissue techniques, and neural mobilization, which are commonly used in the management of MSDs such as low back pain and osteoarthritis [12,13]. Evidence suggests that manual therapy may improve pain, range of motion, and function in selected conditions [14], and cost-effectiveness analyses indicate it may be more economical than usual general practitioner care for some musculoskeletal conditions [15,16]. In dance medicine, manual therapy is widely used by practitioners to address joint restrictions and movement impairments [17]; however, the literature specifically examining its use in dancers remains sparse [17,18].

Similarly, Pilates Method rehabilitation refers to the clinical application of Joseph Pilates’ method, emphasizing core control, spinal alignment, and functional breathing. Its emphasis on core stability, controlled movement, flexibility, and mind–body integration aligns closely with the biomechanical and aesthetic demands of dance [19,20]. Historically, the biomechanical rationale for utilizing Pilates apparatuses in dance-specific movements has been well-documented; for instance, early kinematic analyses of the demi-plié on the Reformer provided a physiological foundation for its use as a highly specific functional conditioning and rehabilitation tool [21]. The method is grounded in six key principles—centering, concentration, control, precision, flow, and breath, which collectively aim to enhance neuromuscular efficiency and optimize movement quality [22,23]. Evidence indicates that core strengthening using the Pilates Method improves multiple components of physical fitness relevant to dancers, including trunk strength, muscular endurance, balance, and proprioception [24,25]. Pilates is also widely used in rehabilitation settings for dancers recovering from overuse injuries, offering options that can be progressively tailored to the dancer’s tolerance and functional goals [19,26].

### 1.3. Context of Rehabilitation in Dance Medicine

Rehabilitation for MSDs in dancers traditionally encompasses a wide range of interventions, from progressive loading protocols for tendinopathies [27] to pharmacological management and, in severe cases, surgical intervention [28]. While emerging technological innovations, such as AI-assisted systems and robotic rehabilitation, are showing promise in general clinical settings [29], their practical application in the dance community remains limited and largely unexplored. This review focuses specifically on manual therapy, core-stability training, and Pilates, as these modalities are deeply integrated into dance culture and clinical practice, yet they lack a systematic mapping of their evidence base within this high-demand population.

### 1.4. Rationale and Objectives

Despite the widespread clinical use of manual therapy and Pilates in dance rehabilitation, the conceptual distinctions between general performance enhancement, injury prevention, and targeted clinical rehabilitation are often blurred in the literature. Furthermore, existing studies tend to focus on isolated interventions, small samples, or non-dancer populations.

A scoping review methodology was therefore chosen over a systematic review, as it allows for the comprehensive mapping of a diverse and heterogeneous body of literature to identify existing knowledge gaps. Guided by the Population–Concept–Context (PCC) framework, the primary objective of this scoping review is to systematically identify, categorize, and synthesize the existing evidence regarding joint manipulation, mobilization, core-focused training, and interventions grounded in the Pilates Method in adult dancers with musculoskeletal disorders. By integrating evidence from dance medicine and rehabilitation science, this review aims to provide a comprehensive overview of current approaches and support evidence-informed rehabilitation strategies.

## 2. Materials and Methods

### 2.1. Study Design

This scoping review was conducted following the methodological framework originally proposed by Arksey and O’Malley [30] and subsequently refined by Levac et al. [31]. The reporting of the review adheres to the Preferred Reporting Items for Systematic Reviews and Meta-Analyses extension for Scoping Reviews (PRISMA-ScR) [32]. The protocol for this scoping review was preregistered on the Open Science Framework (OSF) on 24 January 2026 (https://doi.org/10.17605/OSF.IO/J4NRS). A scoping review methodology was deliberately chosen over a systematic review because the primary objective was to map a broad, diverse, and heterogeneous body of literature regarding musculoskeletal rehabilitation in dancers. Identifying knowledge gaps and mapping key concepts across various dance styles and intervention types was deemed more appropriate at this stage than answering a single clinical question on efficacy.

No deviations from the preregistered protocol occurred during the conduct of this scoping review.

### 2.2. Eligibility Criteria (PCC Framework)

Eligibility criteria were strictly guided by the Population–Concept–Context (PCC) framework [33] to ensure a transparent approach for defining the scope of the review.

In dance medicine, the boundary between late-stage rehabilitation and secondary injury prevention is inherently blurred. Interventions focusing on neuromuscular efficiency, biomechanical correction, and specific conditioning are essential components of the rehabilitation continuum to prevent injury recurrence. Therefore, studies addressing secondary prevention and biomechanical correction in dancers were considered aligned with the PCC framework’s context of musculoskeletal disorder management.

The detailed inclusion and exclusion criteria applied during the screening process are systematically summarized in Table 1.

Peer-reviewed primary research studies of any design (Randomized Controlled Trials, intervention studies) were included. The deliberate inclusion of lower-level evidence, such as case reports and case series, was deemed necessary and is explicitly justified due to the nascent state of specific dance medicine research. In high-demand populations like dancers, highly individualized clinical protocols are frequently first documented in case studies before being tested in larger trials.

### 2.3. Search Strategy

To ensure high reproducibility and comprehensive coverage, a systematic search was conducted across six major electronic databases: PubMed, Scopus, Web of Science, SPORTDiscus, CINAHL, and PEDro, covering the literature from database inception up to February 2026. Only English-language peer-reviewed studies were considered. The full database-specific search strategies used for each electronic database are provided in Appendix A. The exact Boolean string applied across all databases was: (“manual therapy” OR “manipulative therapy” OR mobilization OR “soft tissue therapy” OR “core training” OR “core stability” OR “core-based exercise” OR “core strengthening” OR Pilates OR “Pilates method”) AND (“musculoskeletal disorder” OR “musculoskeletal pain” OR “overuse injur*” OR injury OR rehabilitation) AND (dancer* OR “dance student*” OR “professional dance” OR ballet OR “contemporary dance”).

### 2.4. Study Selection

All records identified through the database searches were imported into a reference management system, where duplicates were automatically and manually removed. To minimize selection bias, the screening process was conducted blindly. Two independent reviewers screened the titles and abstracts of all unique records against the predefined PCC criteria. Studies that appeared relevant were advanced to full-text review, which was independently assessed by the same two reviewers. Any disagreements regarding study inclusion were resolved through comprehensive discussion until a consensus was reached; when necessary, a third senior reviewer was consulted.

### 2.5. Data Extraction and Synthesis

**Figure 1:**
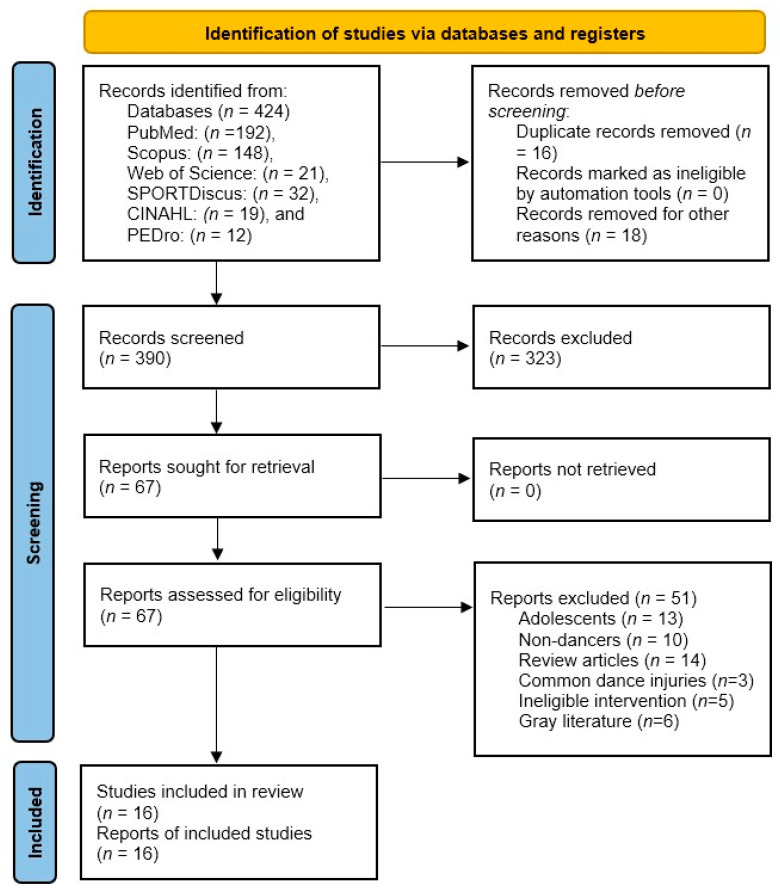
PRISMA-ScR flow diagram illustrating the study selection process. A total of 424 records were identified through database searching (PubMed: 192, Scopus: 148, Web of Science: 21, SPORTDiscus: 32, CINAHL: 19, and PEDro: 12) After removing duplicates and screening records, full-text eligibility was assessed. Ultimately, 16 studies met the inclusion criteria and were incorporated into the final synthesis.

Data extraction was performed independently by two reviewers using a standardized and piloted form. Extracted data included author(s), year of publication, study design, participant demographics (dance genre and performance level), musculoskeletal condition, specific intervention details (e.g., techniques, dosage), and main clinical outcomes. Consistent with the scoping review methodology described by Peters et al. [34] and the PRISMA-ScR guidelines, a formal critical appraisal (risk-of-bias assessment) of the included studies is not mandatory, as the aim is to map the extent of evidence regardless of methodological quality. However, major methodological limitations of the literature are addressed in the discussion. Extracted data were synthesized descriptively and presented both narratively and in tabular form. The entire study selection process, from initial identification to final inclusion, is visually summarized in the PRISMA-ScR flow diagram (Figure 1).

## 3. Results

A total of 16 studies met the eligibility criteria and were included in the final synthesis. All studies involved adult, professional, or pre-professional dancers presenting with musculoskeletal conditions requiring rehabilitative intervention or targeted injury management. The included studies comprised a diverse range of evidence levels: one randomized controlled trial (RCT), three intervention studies, three case series, and nine case reports.

The musculoskeletal conditions mapped across the literature were highly heterogeneous, including sacroiliac joint pain, chronic ankle instability, flexor hallucis longus (FHL) stenosing tenosynovitis, acetabular labral tears, post-surgical hip and ankle dysfunction, low back pain, chronic cervicalgia, patellofemoral pain syndrome, and post-acute sequelae of SARS-CoV-2. The documented interventions were categorized into three broad modalities: manual therapy and joint manipulation, core stability/motor control training, and rehabilitation grounded in the Pilates Method.

The main characteristics of the included studies, detailing study design, participant demographics (dance style and performance level), musculoskeletal condition, intervention type, and reported outcomes—are systematically summarized in Table 2.

### 3.1. Manual Therapy and Joint Manipulation Approaches

Manual therapy was the most frequently mapped intervention category, represented in over half of the included studies. These studies described the clinical application of hands-on therapeutic techniques to address pain, joint dysfunction, and movement impairments in dancers. Mulligan Mobilizations with Movement were applied in dancers with sacroiliac joint pain and lower-limb overuse symptoms, with studies reporting reductions in pain intensity and improvements in functional movement patterns. Multimodal manual therapy approaches, typically including joint mobilization, soft tissue techniques, and postural retraining, were employed for spinal conditions such as chronic cervicalgia and thoracic spine pain.

More recent literature mapped the use of manual therapy in specialized post-surgical and severe injury rehabilitation contexts. Curley et al. [48] documented a structured manual therapy protocol following hip arthroscopy, observing restoration of range of motion and return to dance progression. Similarly, Khoo-Summers & Bloom [50] mapped the conservative management of acetabular labral tears using joint mobilizations. Furthermore, innovative manual techniques were identified in the literature, such as Instrument-Assisted Soft Tissue Mobilization (IASTM) combined with blood flow restriction training, which was applied to international standard dancers with chronic ankle instability [45]. Across these diverse conditions, manual therapy was frequently described as a supportive component for restoring dance-specific functional capacity.

### 3.2. Core Stability and Motor Control Interventions

Core-focused interventions, emphasizing the stabilization of deep trunk musculature and motor control training, were mapped across several study designs, including high-level evidence. One randomized controlled trial (RCT) was identified in this domain. Vera et al. [46] investigated a comprehensive, core-inclusive injury prevention and management program in professional ballet dancers, documenting a significant reduction in injury incidence and severity.

In lower-level evidence studies, stabilization exercises and movement reeducation were mapped in the management of focal degenerative joint disease of the spine in an adult dancer [35], while targeted core strength training was utilized for low back pain in pre-professional dancers. Interventions such as dynamic sling systems and targeted home-based core exercises reported improvements in trunk stability and pain reduction [43]. These findings highlight the critical role of core-focused rehabilitation strategies in addressing lumbo-pelvic dysfunction and optimizing the kinetic chain in dance populations.

### 3.3. Pilates-Based Interventions

A distinct subset of the mapped literature specifically investigated rehabilitation grounded in the Pilates Method, aligning closely with the biomechanical demands of dance. The application of specialized Pilates apparatus was documented by Che et al. [47], who investigated a 12-week Pilates Reformer training program in Latin dancers, reporting enhanced biomechanical control and improved technical execution of complex movements. Additionally, Panhan et al. [49] mapped the neuromuscular effects of Pilates Mat exercises in ballet, reporting improved neuromuscular efficiency of the multifidus and internal oblique muscles. While less frequently represented as standalone clinical interventions compared to manual therapy, these studies illustrate the emerging integration of Pilates as a therapeutic tool for neuromuscular retraining and injury management in dancers.

### 3.4. Summary of Patterns Across Studies

The overall pattern of findings indicates that manual therapy and core training approaches (including Pilates) are deeply embedded in the clinical management of MSDs in dancers. Notably, the evidence base appears to be transitioning. While the historical literature is heavily dominated by isolated case reports and case series, more recent publications map the introduction of a randomized controlled trial (RCT) and structured intervention studies. Across the entire body of evidence, studies consistently reported positive clinical outcomes, including reductions in pain, improvements in joint mobility, and enhanced return-to-performance metrics. However, from a scoping perspective, considerable variability remains in study designs, specific intervention dosages, and outcome measures. The lack of standardized reporting protocols across dance genres limits direct comparability, underscoring a persistent gap in universally established clinical guidelines for dance rehabilitation.

### 3.5. Methodological Quality Summary

In accordance with scoping review methodology, a formal risk-of-bias assessment was not performed; however, a structured qualitative appraisal of the evidence base was conducted to provide transparency regarding the strength of the findings. The majority of the included studies (*n* = 12, 75%) consisted of case reports and case series (Level 4 evidence). While these studies offer high clinical granularity and detail highly individualized dance-specific protocols, they are inherently limited by the absence of control groups and the inability to establish causality.

The emerging body of higher-level evidence, including one Randomized Controlled Trial (RCT) and three intervention studies, demonstrates a positive shift toward more rigorous experimental designs in dance rehabilitation. However, common methodological limitations across the literature include small sample sizes, lack of long-term follow-up, and the use of diverse, non-standardized outcome measures, which limit the ability to perform a meta-analysis. Despite these limitations, the evidence base provides a comprehensive “map” of current clinical practices, effectively highlighting the practical integration of manual therapy and the Pilates Method in the dance community.

## 4. Discussion

Manual therapy remains the most frequently documented approach, with a relatively broad range of techniques described. In contrast, core-focused and Pilates Method rehabilitation, despite their widespread use in clinical and professional dance settings, are transitioning from a state of being virtually absent in the literature to emerging concepts supported by recent empirical studies and a randomized controlled trial (RCT). Collectively, these findings indicate that while rehabilitation practices in dance medicine are diverse and clinically rich, the scientific evidence base is currently undergoing a necessary shift from purely descriptive accounts to structured clinical investigations.

### 4.1. The Established Role of Manual Therapy

Manual therapy represents one of the most established and widely utilized rehabilitation approaches in dance medicine. The literature describes a broad spectrum of hands-on techniques, including spinal manipulation, joint mobilization, soft tissue mobilization, myofascial release, massage, and manual traction, all of which aim to address soft tissue dysfunction, joint restrictions, pain, and impaired movement patterns. These techniques are frequently applied in the management of dance-related injuries due to the high physical demands placed on dancers and the prevalence of overuse conditions affecting the spine, hip, foot, and ankle. Manual therapy is used to improve tissue extensibility, increase joint range of motion, modulate pain, reduce swelling, and restore functional movement patterns essential for dance performance [51,52,53].

Spinal manipulation and mobilization are commonly described in the literature as effective methods for restoring segmental mobility and reducing pain in dancers with spinal or pelvic dysfunction. These techniques involve either high-velocity, low-amplitude thrusts or low-velocity oscillatory movements applied within the joint’s passive range, with the goal of improving joint mechanics and neuromuscular control [54,55,56]. Soft tissue techniques, including massage and myofascial release, are frequently used to address muscular tightness, fascial restrictions, and edema, which are common in dancers due to repetitive loading and high training volume [51,57,58]. Other approaches, such as manual lymphatic drainage, traction, and muscle energy techniques, are applied depending on the stage of injury and the specific tissues involved [59,60,61].

The mechanisms underlying manual therapy are multifactorial. Mechanical effects include increased tissue extensibility and improved joint mobility, while neurological effects involve modulation of proprioceptive and nociceptive input, influencing both peripheral and central pain processing [54,62,63]. Manual therapy may also restore functional movement patterns by addressing compensatory strategies that develop after injury, thereby reducing the risk of recurrence [11]. In acute injury management, manual therapy is often applied away from the injury site to control pain and maintain mobility without disrupting tissue healing. In chronic or recurrent injuries, it is used to address persistent stiffness, pain, and maladaptive movement patterns that predispose dancers to reinjury [54,64].

The recent literature has expanded this scope by mapping structured manual therapy protocols in specialized post-surgical contexts, such as hip arthroscopy [48], as well as conservative management of labral tears [50]. Furthermore, innovative integrations, such as Instrument-Assisted Soft Tissue Mobilization (IASTM) combined with blood flow restriction training [45], demonstrate the modernization of manual interventions. However, while evidence suggests that manual therapy can reduce pain and improve function, many studies rely on extrapolation from athletic or general musculoskeletal research [65,66]. The literature emphasizes that manual therapy is most effective when integrated into a comprehensive rehabilitation program that includes active exercise, neuromuscular retraining, and technique modification [67,68]. Safety considerations include avoiding aggressive soft tissue release in the presence of joint instability and ensuring that practitioners have specialized training in dance medicine [11,69]. Further research is still needed to evaluate its effectiveness in specific dance-related injuries [18,19].

### 4.2. Core Training and Pilates Method Interventions: An Emerging Evidence Base

Core-based and Pilates Method rehabilitation is widely used in dance medicine, particularly for improving neuromuscular control, postural alignment, and movement efficiency. Historically, its use in dance rehabilitation was based primarily on clinical experience and theoretical alignment with dance biomechanics, rather than empirical evidence, with concepts often extrapolated from non-dance populations [70,71,72,73]. The principles of Pilates align with the biomechanical demands of dance, emphasizing trunk-pelvis control and efficient force transfer.

Our review indicates that Pilates Method rehabilitation is emerging but is still dominated by low-level evidence and case studies. However, the introduction of the recent literature marks a pivotal shift. Specifically, the utilization of Pilates apparatus has been documented to enhance the biomechanical control of movement in Latin dancers [47], while Pilates Mat interventions have demonstrated improvements in the neuromuscular efficiency of stabilizing musculature [49].

Similarly, the evidence surrounding broader core stability training has recently been elevated by the inclusion of high-quality designs. A randomized controlled trial by Vera et al. [46] provides critical empirical support, demonstrating that comprehensive, core-inclusive injury prevention and motor control program significantly reduces musculoskeletal complaints and injury severity in professional and student dancers. Despite this progress, the literature traditionally relied on studies from non-dance populations [74,75,76,77], and access to specialized Pilates Method rehabilitation resources varies widely, with larger dance companies more likely to offer multidisciplinary care compared to smaller organizations [70]. Comparisons with broader clinical literature reveal that Pilates demonstrates benefits in non-dance populations [78,79,80,81,82], but the unique biomechanical demands of dance necessitate further dancer-specific controlled trials [19].

### 4.3. Methodological Considerations and Heterogeneity

A primary observation of this review is the significant heterogeneity regarding dance genres, injury types, and intervention protocols. The physical demands of classical ballet differ fundamentally from contemporary, modern, or Latin dance, yet the literature often treats ‘dancers’ as a monolithic group. This lack of stratification, combined with the absence of standardized core-training and Pilates protocols, complicates the synthesis of results and limits their generalizability. Detailed reporting of intervention dosages and dancer-specific adaptations remains inconsistent across the mapped literature.

### 4.4. The Role of Case Reports and Emerging RCTs in Elite Populations

The predominance of case reports and case series (12 out of 16 studies) reflects a known challenge in dance medicine: the difficulty of recruiting large, homogenous samples of elite performers for adequately powered clinical trials. While case reports offer lower-level evidence, they provide granular, clinical insights into highly individualized treatment pathways, a necessity in professional dance where ‘one-size-fits-all’ rehabilitation is often ineffective. Historically, this created a ‘circular’ lack of evidence where valuable clinical expertise remained undocumented in high-level trials. However, the recent emergence of an RCT [46] and structured intervention studies indicates that the field is maturing, bridging the gap between clinical practice and rigorous scientific methodology.

### 4.5. Implications for Future Research

For clinicians, the current evidence suggests that while Manual Therapy and Pilates are staples in the studio, their application should be guided by specific biomechanical analysis rather than tradition alone. Future research must continue to move beyond descriptive case studies. We propose the development of standardized reporting checklists for core and Pilates Method interventions and the implementation of N-of-1 trials or multi-center RCTs that account for the artistic and mechanical specificities of different dance styles. Longitudinal studies examining return-to-dance outcomes, recurrence rates, and objective biomechanical assessments (such as motion analysis and electromyography) would provide valuable insights into the long-term effectiveness of these interventions.

### 4.6. Limitations

This review has several limitations. While improving, the limited number of high-level studies on core-focused and Pilates Method rehabilitation in dancers restricts the breadth of evidence that could be synthesized. The heterogeneity of study designs, outcome measures, and reporting quality further restricts the ability to draw definitive conclusions. The exclusion of non-injured dancers and performance-enhancement studies, while necessary to maintain conceptual clarity (PCC framework), reduced the number of eligible studies. Finally, the reliance on published peer-reviewed literature may have excluded unpublished protocols used in professional dance settings, suggesting that the true extent of rehabilitation practices in dance medicine may be underrepresented.

## 5. Conclusions

This scoping review synthesized the available evidence on manual therapy, core-based training, and interventions grounded in the Pilates Method for adult dancers with musculoskeletal injuries. Manual therapy emerged as the most frequently described intervention, reflecting its long-standing role in addressing pain, joint restrictions, and soft-tissue dysfunction in dance populations. However, while its clinical utility is evident, the supporting evidence remains heavily reliant on case reports, case series, or extrapolation from broader musculoskeletal research.

Core-based and Pilates Method rehabilitation approaches, previously applied primarily in conditioning rather than rehabilitation contexts, are now emerging within the clinical literature. Specifically supported by a recent randomized controlled trial and targeted intervention studies, these modalities demonstrate potential benefits for lumbo-pelvic dysfunction, neuromuscular efficiency, and biomechanical control. Nevertheless, the overall evidence base remains narrow, methodologically heterogeneous, and constrained by small sample sizes.

Overall, the findings indicate that while rehabilitation practices in dance medicine remain heavily shaped by clinical expertise and tradition, the field is beginning a necessary transition toward empirical validation. Advancing the field will require well-designed clinical studies, detailed reporting of intervention parameters, and objective assessments tailored to the specific biomechanical and technical demands of different dance genres. Strengthening the evidence base for manual therapy, core-based training, and the emerging clinical application of Pilates will support the development of standardized, dancer-specific protocols, ultimately improving injury outcomes and supporting the long-term health and performance of dancers.

## Figures and Tables

**Table 1:** Inclusion and exclusion criteria based on the Population–Concept–Context (PCC) framework.

Framework	Inclusion Criteria	Exclusion Criteria
Population (P)	Adult dancers (aged ≥ 18 years) of any skill level (professional, pre-professional, amateur) across all dance genres (e.g., ballet, contemporary, modern, Latin).	Studies focusing exclusively on children/adolescents (under 18) or non-dancer populations (unless dancer-specific data are reported separately).
Concept (C)	Rehabilitation interventions specifically involving Manual Therapy techniques (e.g., joint mobilization, manipulation, IASTM), Core-stability, or the Pilates Method.	Interventions used solely for general fitness, aesthetic performance enhancement without an injury context, or modalities completely unrelated to manual/core/Pilates.
Context (C)	Clinical, sports, performing arts, or educational environments focused on injury management, post-surgical rehabilitation, or rehabilitation of musculoskeletal disorders (MSDs).	Literature without a clinical or rehabilitative focus; grey literature, dissertations, theses, conference abstracts, non-peer-reviewed sources, and non-English studies.

**Table 2:** Summary of included studies mapping study design, dancer population, specific condition, rehabilitative intervention, and reported outcomes.

Study	Study Design	Population and Condition	Intervention	Main Outcomes
Hagins [35]	Case report	Adult dancer with focal degenerative joint disease of the spine	**Type:** Stabilization exercises **Components:** Movement reeducation	Observed pain reduction Improved spinal function
Krzyzanowicz et al. [36]	Case series	Recreational adult dancers with sacroiliac joint pain	**Type:** Mulligan Concept **Components:** Mobilizations with Movement	Observed decreased SIJ pain Improved function
Wentzell [37]	Case report	Pre-professional ballet dancer with chronic recurrent FHL stenosing tenosynovitis	**Type:** Conservative management **Components:** Manual therapy and exercise	Observed symptom reduction Restored pointe work
Masaracchio et al. [38]	Case report	Adult dancer with thoracic spine pain	**Type:** Multimodal PT **Components:** Manual therapy and movement training	Observed pain reduction Improved thoracic mobility
Aguiar et al. [39]	Intervention study	Classical ballet dancers with lower-limb pain	**Type:** Manual therapy **Components:** Mulligan Concept mobilizations	Observed reduced pain Improved ROM
Brown & Disney [40]	Case report	Adult dancer with chronic cervicalgia	**Type:** Multimodal physical therapy	Observed decreased neck pain Improved cervical function
Ojofeitimi [41]	Case report	Professional dancer with second MTP joint instability	**Type:** Conservative management **Components:** Manual therapy and taping	Observed improved joint stability Return to performance
Schaeffer & Harary [42]	Case report	Professional modern dancer with post-acute sequelae of SARS-CoV-2	**Type:** Manual therapy-based rehabilitation	Observed improved endurance Enhanced functional capacity
Kline and Kraus [43]	Case series	Pre-professional ballet dancers with low back pain	**Type:** Core strength training **Components:** Home exercises and sling system	Observed reduced low back pain Improved core function
Welsh et al. [44]	Case report	Adult female dancer with patellofemoral pain syndrome	**Type:** Regional interdependence-based rehab **Components:** Manual therapy	Observed pain reduction Improved functional capacity
Liu & Wang [45]	Intervention study	International standard dancers with Chronic Ankle Instability (CAI)	**Type:** IASTM **Components:** Combined with Blood Flow Restriction Training (BFRT)	Observed improved ankle stability Improved strength and proprioception
Vera et al. [46]	RCT	Professional ballet dancers (Injury prevention/management)	**Type:** Comprehensive Injury Prevention Program **Components:** Core and functional training	Associated with a reduction in injury incidence and severity
Che et al. [47]	Intervention study	Latin dancers (Biomechanical technique dysfunction)	**Type:** Pilates Reformer training **Duration:** 12 weeks	Observed enhanced biomechanical control of movement
Curley et al. [48]	Case series	Professional ballet dancers post-Hip Arthroscopy	**Type:** Post-surgical rehab protocol **Components:** Joint mobilizations, manual therapy and progressive loading	Observed successful return to ballet progression Return to performance
Panhan et al. [49]	Case report	Ballet dancer (Neuromuscular efficiency/Injury prevention focus)	**Type:** Pilates Method **Components:** Mat exercises	Observed improved neuromuscular efficiency of multifidus/obliques
Khoo-Summers & Bloom [50]	Case report	Professional ballet dancer with acetabular labral tear	**Type:** Manual therapy and neuromuscular retraining **Components:** Joint mobilizations	Associated with avoidance of surgery Pain resolution Full return to performance

Note: SIJ = sacroiliac joint; FHL = flexor hallucis longus; PT = physical therapy; ROM = range of motion; MTP = metatarsophalangeal; RCT = Randomized Controlled Trial; IASTM = Instrument-Assisted Soft Tissue Mobilization. Bold text highlights the categorization of interventions into ‘Type’ (general approach) and ‘Components’ (specific methods used).

## Data Availability

No new data were created or analyzed in this study.

## Abbreviations

The following abbreviations are used in this manuscript:

MSDs	Musculoskeletal Disorders
PRISMA-ScR	Preferred Reporting Items for Systematic Reviews and Meta-Analyses extension for Scoping Reviews
OSF	Open Science Framework
PCC	Population–Concept–Context (framework of the Joanna Briggs Institute)
PT	Physical Therapy/Physical Therapist (Table 1: “Multimodal PT”)
SIJ	Sacroiliac Joint
FHL	Flexor Hallucis Longus
MTP	Metatarsophalangeal
RCT	Randomized Control Trial
IASTM	Instrument-Assisted Soft Tissue Mobilization

## Appendix A. Full Database-Specific Search Strategies

- PubMed (MEDLINE): (“manual therapy”[MeSH] OR “manipulative therapy” OR “mobilization” OR “soft tissue therapy” OR “core training” OR “core stability” OR “Pilates”[MeSH] OR “Pilates method”) AND (“musculoskeletal diseases”[MeSH] OR “musculoskeletal pain” OR “overuse injury” OR “rehabilitation”[MeSH]) AND (“dancing”[MeSH] OR “dancer*” OR “ballet”)
- Scopus: TITLE-ABS-KEY (“manual therapy” OR “manipulative therapy” OR mobilization OR “soft tissue therapy” OR “core training” OR “core stability” OR “core-based exercise” OR “core strengthening” OR Pilates OR “Pilates method”) AND TITLE-ABS-KEY (“musculoskeletal disorder” OR “musculoskeletal pain” OR “overuse injur*” OR injury OR rehabilitation) AND TITLE-ABS-KEY (dancer* OR “dance student*” OR “professional dance” OR ballet OR “contemporary dance”)
- Web of Science (Core Collection): TS = (“manual therapy” OR “manipulative therapy” OR mobilization* OR “soft tissue therapy” OR “core training” OR “core stability” OR “Pilates” OR “Pilates method”) AND TS = (“musculoskeletal disease*” OR “musculoskeletal pain” OR “overuse injur*” OR rehabilitation) AND TS = (dancer* OR ballet OR “dance student*”)
- SPORTDiscus (EBSCOhost): TX (“manual therapy” OR “manipulative therapy” OR mobilization* OR “soft tissue therapy” OR “core training” OR “core stability” OR “Pilates”) AND TX (“musculoskeletal disease*” OR “musculoskeletal pain” OR “overuse injur*” OR rehabilitation) AND TX (dancer* OR ballet OR “dance student*”)
- CINAHL (EBSCOhost): (MH “Manual Therapy” OR “manipulative therapy” OR mobilization* OR “core training” OR “core stability” OR MH “Pilates”) AND (MH “Musculoskeletal Diseases” OR “musculoskeletal pain” OR “overuse injury” OR MH “Rehabilitation”) AND (MH “Dancing” OR dancer* OR ballet)
- PEDro: Note: Due to PEDro’s character limits and restricted search interface, a simplified keyword strategy was utilized in the Abstract & Title field: (dance* OR ballet) AND (Pilates OR “manual therapy” OR mobilization OR core)
